# Genotype data for single nucleotide polymorphism markers in sporadic breast cancer related genes in a Sri Lankan case–control cohort of postmenopausal women

**DOI:** 10.1186/s13104-019-4472-0

**Published:** 2019-07-19

**Authors:** Nirmala Dushyanthi Sirisena, Nilakshi Samaranayake, Vajira H. W. Dissanayake

**Affiliations:** 1 Human Genetics Unit, Faculty of Medicine, University of Colombo, No. 25 Kynsey Road, Colombo 8, Sri Lanka; 2 Department of Parasitology, Faculty of Medicine, University of Colombo, Colombo 8, Sri Lanka

**Keywords:** Breast cancer, Genotypes, Postmenopausal, Single nucleotide polymorphisms

## Abstract

**Objective:**

The data presented herein represents the raw genotype data of a recently conducted larger study which investigated the association of single nucleotide polymorphisms (SNPs) in breast cancer related genes with the risk and clinicopathological profiles of sporadic breast cancer among Sri Lankan women. A case–control study design was adopted to conduct SNP marker disease association testing in an existing blood resource obtained from a cohort of Sri Lankan postmenopausal women with clinically phenotyped sporadic breast cancer and healthy postmenopausal women. The list of haplotype-tagging SNP markers for genotyping was selected based on information available in the published literature and use of bioinformatics tools and databases. Genotyping of 57 selected SNPs in 36 breast cancer related genes was performed using the iPLEX Sequenom Mass-Array platform.

**Data description:**

The raw genotype data for the 57 SNPs genotyped in 350 women with breast cancer and 350 healthy women are presented in this article. This data might be relevant to other researchers involved in investigating the role of SNPs in breast cancer related genes with the risk of sporadic breast cancer in South Asian populations.

## Objective

Breast cancer accounts for approximately 23% of all cancers in females and 12% of all cancers among Sri Lankans. Notably, 62.1% of breast cancers are diagnosed in Sri Lankan women aged above 50 years [1]. Herein we present the raw genotype data of a recently published case–control study, in which 350 Sri Lankan postmenopausal women with invasive breast cancer (cases) and 350 healthy postmenopausal women (controls) were genotyped for 57 haplotype-tagging single nucleotide polymorphisms (SNPs) in 36 candidate genes associated with sporadic breast cancer using iPLEX Sequenom Mass-Array platform. The study population was from all over the country, minimizing potential selection bias. This cohort was genotyped to identify the association of common genetic variants with the risk and clinicopathological profiles of sporadic breast cancer. SNPs in candidate breast cancer genes with minor allele frequencies above 0.05 in the Gujarati Indians in Houston, USA (GIH) were obtained from the International HapMap Project database. GIH were the only South Asian population group in the HapMap project or other similar projects with dense genotypes available at the time of study design. The methods used in selecting the SNP markers have been described in previous publications [2, 3]. Results showed that *XRCC2:*rs3218550 and *PHB:*rs6917 were associated with increased risk. *CDH1*:rs13689 and *ATM*:rs1801516 were found to be protective [2]. The clinical characteristics of this cohort were reported in a previous publication [3]. SNPs in the *AKT1*, *BRCA1*, *BRCA2*, *CCND1*, *CDH1* and *NQO2* genes were associated with different clinicopathological profiles of breast cancer [3]. The functional effects of *XRCC2*:rs3218550 and *PHB*:rs6917 were further investigated using the dual-luciferase assays [4].

The raw genotype data might be relevant to other researchers involved in investigating the association of SNPs in breast cancer related genes with sporadic breast cancer risk in South Asian populations.

## Data description

DNA was extracted using the Promega Wizard^®^ Genomic DNA purification kit and quantified using the Quantus fluorometer with QuantiFluor^®^ double stranded DNA system according to the manufacturer’s protocol (Promega, Madison, USA). Each sample was diluted in distilled water and normalized to a DNA concentration of 10.0 ng/μl.

Genotyping was done using the Agena Bioscience MassArray technology on a Compact Spectrometer, iPLEX GOLD chemistry (Australian Genome Research Facility, Gehrmann Laboratories, University of Queensland, Australia) [5]. Primers flanking the gene region containing the SNPs were designed using MassArray Designer software. All samples (10 ng/µl) were transferred into 384 well polymerase chain reaction (PCR) plates for genotyping.

Samples were amplified from a 5 µl final PCR volume composed of 1 × PCR buffer, 2 mM MgCl_2_, 500 µM deoxynucleotide triphosphates (dNTPs), 0.1 µM each PCR primer, 0.5 U of HotStarTaq enzyme, and 1 µl DNA. The thermal cycling conditions included a first denaturation step at 95 °C for 2 min, followed by 45 cycles of denaturation at 95 °C for 30 s, annealing at 56 °C for 30 s, and extension at 72 °C for 1 min, with a final extension step at 72 °C for 5 min. To neutralize unincorporated dNTPs, PCR products were treated with 0.5 U shrimp alkaline phosphatase by incubation at 37 °C for 40 min, followed by enzyme inactivation by heating at 85 °C for 5 min. By adding 2 µl of an iPLEX Gold extension reaction cocktail to the purified PCR products, the extension reaction was carried out in a final volume of 9 µl containing 0.222 × iPLEX buffer, 1 × iPLEX termination mix, 1 × iPLEX enzyme, and the SBE primer mix of extension primers. The iPLEX extension reaction was performed as follows: initial denaturation step at 94 °C for 30 s, followed by 40 cycles of a denaturation step at 94 °C for 5 s, 5 cycles of annealing at 52 °C for 5 s and extension at 80 °C for 5 s and a final extension step at 72 °C for 3 min. After desalting of the products by using SpectroCLEAN resins following the manufacturer’s protocol, cleaned extension products were dispensed onto a 384 SpectroCHIP array using an RS1000 Nanodispenser, and the array was introduced into a MassARRAY Compact mass spectrometer. Spectra were acquired using SpectroAcquire software and data analysis, including automated allele calling, was done using MassARRAY Typer software, version 4.0.5. Fifty-seven SNPs were successfully genotyped, and the average SNP call rate was 99.87% in both cases and controls.

The raw genotype data for the 57 SNPs genotyped in the 350 cases and 350 controls are shown in data files 1 and 2 respectively and the primer sequences are included in data file 3 in Table 1 [6].

**Table 1:** Overview of data files

Label	Name of data file	File types (file extension)	Data repository and identifier (DOI or accession number)
Data file 1	Raw genotype data for the postmenopausal women with sporadic breast cancer	MS Excel file (.xlsx)	Figshare [10.6084/m9.figshare.7159514] [6]
Data file 2	Raw genotype data for the healthy postmenopausal women	MS Excel file (.xlsx)	Figshare [10.6084/m9.figshare.7159514] [6]
Data file 3	Primer sequences	MS Excel file (.xlsx)	Figshare [10.6084/m9.figshare.7159514] [6]

## Limitations

- The selected set of SNPs may not give as comprehensive a view of genetic variation as genomic sequencing does.
- It is possible that SNPs which show a null association either do not modify the susceptibility to breast cancer or their effects are minimal and can be detected only with larger study samples.
- These SNPs are mainly low-penetrant alleles that probably exert their effects through complex gene–gene and/or gene-environment interactions. Such interactions were not investigated in this study.

## Data Availability

The datasets generated and/or analyzed during the current study are available in the Figshare repository [10.6084/m9.figshare.7159514] [6]

## Abbreviations

dNTPs: deoxynucleotide triphosphates
GIH: Gujarati Indians in Houston, USA
PCR: polymerase chain reaction
SNP: single nucleotide polymorphisms
